# Supplementary material for: Using stakeholder insights to enhance engagement in PhD professional development
Source: PLoS One. 2022 Jan 27;17(1):e0262191. doi: 10.1371/journal.pone.0262191 (PMC8794081; doi:10.1371/journal.pone.0262191)
Supplement: S1 Table — (PDF) [file pone.0262191.s001.pdf]

S1 Table: Number of interviews conducted per stakeholder subgroup, by interviewer

Interviews	Interviewer 1	Interviewer 2	Interviewer 3	Interviewer 4	Interviews per stakeholder subgroup
Pre- and Postdoctoral Researchers	2	2	2	3	**9**
Faculty/ Admin	2	1	2	3	**8**
External-Facing Staff	2	2	3	5	**12**
External Partners: Non-profit/Society	0	2	6	0	**8**
External Employers: Industry	0	3	3	2	**8**
**Total Interviews**	**6**	**11**	**16**	**13**	**45**
