# Supplementary material for: Using stakeholder insights to enhance engagement in PhD professional development
Source: PLoS One. 2022 Jan 27;17(1):e0262191. doi: 10.1371/journal.pone.0262191 (PMC8794081; doi:10.1371/journal.pone.0262191)
Supplement: S2 Table — (PDF) [file pone.0262191.s002.pdf]

S2 Table: Pseudonym assignments for stakeholder interviews

Pre- and Postdoctoral Researchers	**F**aculty and/or Administrators	External facing **S**taff	**E**xternal Partners (Non-Profits/Society)	External Employers (**I**ndustry)
G1-Gaynelle	F1-Fawn	S1-Shandra	E1-Ellen	I1-Iris
G2-Gunnar	F2-Fyodor	S2-Soren	E2-Enzo	I2-Ian
G3-Guangli	F3-Fabayo	S3-Sidone	E3-Emily	I3-Irina
G4-Gaston	F4-Fritz	S4-Santiago	E4-Eduardo	I4-Ivan
G5-Gael	F5-Finley	S5-Sahana	E5-Ellis	I5-Ivy
G6-Gerry	F6-Frank	S6-Sven	E6-Eric	I6-Ilyich
G7-Glenn	F7-Fariba	S7-Shanice	E7-Ebony	I7-Imani
G8-Gus	F8-Fabio	S8-Scott	E8-Evan	I8-Ira
G9-Gretel		S9-Saachi		
		S10-Simha		
		S11-Shyla		
		S12-Sree		
