# Supplementary material for: Using stakeholder insights to enhance engagement in PhD professional development
Source: PLoS One. 2022 Jan 27;17(1):e0262191. doi: 10.1371/journal.pone.0262191 (PMC8794081; doi:10.1371/journal.pone.0262191)
Supplement: S1 File — (PDF) [file pone.0262191.s003.pdf]

S1 File: Rationale and Sample Questions for Stakeholders

Project Rationale

In order to provide resources to pre- and post-doctoral researchers, we first need to determine whether our current understanding of 'the value of a PhD' is accurate, from the point of view of stakeholder groups; perhaps we are missing important aspects/beliefs that have not been adequately appreciated or explored. We will approach stakeholders and pose a specific set of questions that explore their relationship and vision of interacting with academia and, specifically, pre- and post-doctoral researchers and how we can better support those interactions. What we learn from them will help us to better support and develop relevant resources for our pre- and post-doctoral researchers.

Stakeholder Groups and Questions

Internal stakeholders (Stakeholders 1 and 2):

a) Pre- and post-doctoral researchers

b) Faculty

c) Academic administration

<u>Sequence of conversation and questions:</u>

i) Introduction: reminder of purpose

ii) Do you believe that career & professional development for pre- and post-doctoral researchers is a valuable use of time? Why or why not?

(1) If yes (or it depends), how much time is optimal?

(2) If no, what data would convince you that it was a good use of time?

(3) What type of programming do you think would be (most) useful?

iii) Are you aware of examples where career and professional development is working well? (here or elsewhere)

(1) If yes, what is working at institutions that have successfully launched and maintained career development offices/programs?

iv) Do you have other thoughts to add?

v) Thank you for your time!

vi) We may use themes of this to present, no identifying information will be included, do we have your permission to include themes we discussed here today?

External-facing staff (Stakeholder 3):

a) Business Development

b) Alumni Relations

c) Industry Engagement

d) Tech Transfer

<u>Sequence of conversation & questions:</u>

i) Introduction: reminder of purpose

ii) Which external groups do you typically interact with in your role?

iii) What is the intention/purpose of your majority interaction with external stakeholders in your role?

iv) What are the interest areas of the external stakeholders with whom you primarily interact with?

v) Do the people you interact with have an interest in STEM pre- and post-doctoral researchers?

vi) Do you have other thoughts to add?

vii) Thank you for your time!

viii) We may use themes of this to present, no identifying information will be included, do we have your permission to include themes we discussed here today?

External stakeholders (Stakeholders 4 and 5):

a) Societies

b) Funding agencies

c) Employers (all types, ex: business & administration, communications, industry research, law, policy & outreach, government)

Sequence of conversation & questions:

i) Introduction: reminder of purpose

ii) What types of engagement are already in place?

iii) What would make you feel engaged as a partner with an academic institution? Are there modes of interaction that have not been explored or could operate better?

iv) What resources to you believe you have to offer to academic pre- and post-doctoral researchers and faculty? Are there any that we've been missing?

v) How can our pre- and post-doctoral researchers better prepare for entry into your industry?

vi) What would make you want to visit campus to interact directly with our population? vii) Do you follow the national conversation about career development and outcomes of PhD-trained scientists?

viii) Thank you for your time!

ix) We may use themes of this to present, no identifying information will be included, do we have your permission to include themes we discussed here today?
