# Supplementary material for: Using stakeholder insights to enhance engagement in PhD professional development
Source: PLoS One. 2022 Jan 27;17(1):e0262191. doi: 10.1371/journal.pone.0262191 (PMC8794081; doi:10.1371/journal.pone.0262191)
Supplement: S2 File — (PDF) [file pone.0262191.s004.pdf]

S2 File: Sample Wording for Invitation to participate

Sample Wording for Detailed Invitation

NOTE: any italicized content should be tailored to your project

(insert university logo/header here or use templated stationary)

Dear *[INSERT NAME]*,

I hope you are well! I reaching out because I am a member of an NIH Broadening Experience in Scientific Training (BEST) Mastermind Group on Culture Change in Academia around graduate student professional development. I was wondering if I could schedule a time to meet with you to interview you about internal views on *[INSERT STAKEHOLDER SPECIFIC TEXT; e.g., professional development engagement with external stakeholders at our university]*. I have a few questions about how you, in your role engage with *[STAKEHOLDER SPECIFIC TEXT; external stakeholders]*, that should take about 15-20 minutes. Thank you so much for considering the request!

[*FOR PERSONAL CONTACTS* - Insert personal statement here and/or generic – e.g., I'm part of a working group with people in my role across the country, we are hoping to speak with external-facing stakeholders (such as yourself) to see what the need is for companies when it comes to career exploration. I've included more information below; would you be interested in speaking with me more about your experience in your current role?]

[CONTEXT OF PROJECT & POTENTIAL OUTCOMES] In order to provide resources to pre- and post-doctoral researchers, we first need to determine whether our current understanding of 'the value of a PhD' is accurate, from the point of view of multiple stakeholder groups; perhaps we are missing important aspects/beliefs that have not been adequately appreciated or explored. We will approach different stakeholders and pose a specific set of questions that explore

their relationship and vision of interacting with industry and academia and, specifically, with pre- and post-doctoral researchers including how academic institutions and staff can better support those interactions. What we learn from them will help us to better support and develop relevant resources for our pre- and post-doctoral researchers.

Example questions include: *[INSERT STAKEHOLDER-SPECIFIC QUESTIONS HERE]*

- Do the people you interact with have an interest in STEM pre- and post-doctoral researchers?
- Which external groups do you typically interact with in your role?
- What is the intention/purpose of your majority interaction with external stakeholders in your role?
- What are the interest areas of the external stakeholders with whom you primarily interact with?

Best regards,

[INSERT INTERVIEWER NAME and CONTACT INFO]

Sample Wording for Casual Invitation

NOTE: any italicized content should be tailored to your project

(insert university logo/header here or use templated stationary)

Dear *YYY*,

I am [*a member of an NIH Broadening Experiences in Scientific Training Mastermind Group working on graduate student professional development*]. I am reaching out about [*e.g. a research study our group is conducting*] in which I hope you will consider participating.

The purpose of this study is [*to determine whether our current understanding of 'the value of a PhD' is accurate from the point of view of multiple stakeholder groups, (e.g. pre- and post-doctoral researchers, faculty, academic administration, career services offices, tech transfer offices)*]. The group involved in this study are *graduate education professionals across multiple*

universities in the country]. We will [*conduct interviews with specific sets of questions that explore your opinion, relationship, and vision of interactions between industry and academia*]. What we learn will help us to [*better support development of our pre- and post-doctoral researchers for what the workforce needs*]. Interviews will last approximately [*30 minutes*]. [*Our interview will ask about aspects of career and professional development (e.g. what types of engagement are already in place, how can our pre- and post-doctoral researchers better prepare for entry to your industry, what resources you may have to offer)*]. Your feedback is extremely valuable to us, and I would greatly appreciate it if you would be willing to participate. Please let me know if this is of interest to you.

Thank you,

Your name

Contact information
