# Supplementary material for: Using stakeholder insights to enhance engagement in PhD professional development
Source: PLoS One. 2022 Jan 27;17(1):e0262191. doi: 10.1371/journal.pone.0262191 (PMC8794081; doi:10.1371/journal.pone.0262191)
Supplement: S3 File — (PDF) [file pone.0262191.s005.pdf]

S3 File – Underlying de-identified data: Detailed results including example comments

All individuals are referred to by pseudonyms (see Methods).

Stakeholder 1 – Internal Pre- and Post-doctoral Researchers

Support for and perceived benefits of CPD programs identified by pre- and post-doctoral researchers

Theme 1.1: Efficiency, productivity, and content (12 M). Users find CPD affects productivity in a positive way and allows for pre- and post-doctoral researchers to get a broad overview of resources. Glenn elaborated on the need to focus on tools that pre- and post-doctoral researchers can use and apply to their lives currently. Occasional-user Gretel expressed the desire for more pre- and post-doctoral researchers' input in the design of CPD activities. Non-users, such as Gunnar, thought that these events were a waste of time spent on common sense information that could be obtained from their lab more efficiently.

Theme 1.2: Networking, community and role models (11 M). Community-building advantages of professional development activities, e.g. bridging pre- and post-doctoral researchers across labs, were discussed by researchers such as Gaston and Gael. They also noted the benefit of activities that allowed for networking with hiring managers and recruiters rather than only networking with other scientists who do not have influence in non-academic spaces. This theme aligns with findings of a sense of community with pre- and post-doctoral researchers attending professional development training, especially in cohort or mandatory participation models [1]. Some users also mentioned the confidence-building, cultural, and gender-based inclusivity that these programs can provide via intentional conversations and making role models prominent.

Theme 1.3: Exposure and decision-making of career paths (6 M). Some pre- and post-doctoral researchers felt that they needed to decide if a particular career was for them or not. International users, such as Guangli, expressed a need for this programming, citing lack of familiarity with careers in the US. A non-user, Gunnar, who plans an academic career, acknowledged that it is important to understand industry priorities and needs to help them guide their own future pre- and post-doctoral researchers who seek industry positions.

Theme 1.4: Embedded/Required (6 M). Requiring professional development activities in the curriculum would allow for consistent messaging each year of training. Gaston commented that if it is required, then pre- and post-doctoral researchers will need to make time for the activities. Both frequent users and non-users thought embedded programming will provide support and motivation in their career exploration, and this is echoed in a report generated by a separate multi-stakeholder workshop [2]. One of the non-users, Gunnar, pointed to business schools that successfully integrate CPD in curricula. Indeed, many business schools have good examples of externship/internships required for curriculum [3].

Theme 1.5: Prestige- recruitment (4 M). Pre- and post-doctoral researchers feel that having access to offerings can help with their recruitment to companies. Gael succinctly communicated that professional development programs help build a self-fulfilling good reputation for the institution that interests companies, and in turn improves pre- and post-doctoral researcher' track records of landing jobs. Academic-career focused Gunnar also noted that these programs may have unforeseen benefits for pre- and post-doctoral researchers such as facilitating collaboration with industry and promoting translational research.

Opportunities to improve identified by pre- and post-doctoral researchers

Theme 1.6: Consistent exposure throughout training (18 M). Pre- and post-doctoral researchers communicated the desire for consistent exposure to CPD activities throughout training. Gaston, a frequent user as a post-doctoral researcher, mentioned having "rueful regrets" in retrospect about not participating in professional development during PhD training. Many commented that the best approach is regular interactions starting early in training, for even a few hours a month.

Theme 1.7: Centralized access (5 M). Pre- and post-doctoral researchers clearly voiced their desire for centralized programming, as they believe it to be an important and positive feature. Representative across several users, Gael captured this sentiment, noting that centralized CPD programs equalizes access to resources and institutionalizes the concept of career development to facilitate acceptance by faculty. Gael also raised a concern regarding equitable access in the absence of centralized programming; for instance, faculty advisors may not be knowledgeable enough to help direct pre- and post-doctoral researchers interested in careers outside of their own path, or select graduate programs may create separate professional development activities accessed by only their pre- and post-doctoral researchers. Hence professional development programming at the school or university level is more desirable.

Theme 1.8: Growth/challenges (4 M). Pre- and post-doctoral researchers acknowledged that career exploration can be challenging but leads to growth. This theme is captured in Glenn's statement that researchers have to try to push boundaries. Pre- and post-doctoral researchers recognize there are many unknowns with regards to CPD and how it is better to go into the process aware of how hard it will be. Pre- and post-doctoral researchers offered several examples of programming that they think would be most useful, ranging from a condensed and intensive

multi-day workshop to online training such as sessions the NIH Office of Intramural Training and Education (OITE) offers. Another suggestion, repeated in multiple contexts, was that biomedical science programs model professional schools such as Business and Applied Science programs. Some felt that one-on-one coaching was the most efficient approach and a number mentioned specific skills that they felt would be the most useful including people management, budgeting, negotiations, and personal branding.

Theme 1.9: Faculty permission (4 M). Pre- and post-doctoral researchers communicated their concern about faculty buy-in, and, as Glenn suggested, the need to introduce the concept of professional development early to faculty so they are more comfortable with career exploration. Gunnar, a non-user, suggested that faculty approval to participate should not be required. Of note was the sentiment that even help with academic skills such as grant writing assistance from one's mentor should be provided during a structured time, apart from time for other professional development activities.

Stakeholder 2 – Internal Faculty and Administrators

Perceived 'benefits' of CPD identified by Faculty/Administrators

Theme 2.1: Evolving training requirements and climate (12 M). Faculty feel, as Fariba mentioned, that they are living vicariously through their pre- and post-doctoral researchers by being excited for those who are successful in a variety of career paths. The benefit ranged from acknowledgement that training grant applications require a description of career development activities to how participation in these activities can improve mental health. Faculty and administrators acknowledged institutional peer pressure nationally among top tier universities to

provide these opportunities, and that it is the right thing to do for the pre- and post-doctoral researchers.

Theme 2.2: Awareness of workforce outcomes (9 M). Among enthusiastic supporters, there was an acknowledgement that there are limited faculty positions available and that pre- and post-doctoral researchers are choosing a variety of career paths. Frank commented that it is valuable to expose researchers to all one can do with a PhD and that it is helpful to be transparent with workforce outcomes of alumni as examples for current pre- and post-doctoral researchers. However, less supportive respondents believe that professional development is not related to graduate education, that it de-emphasizes academia as a career path, and that individuals who leave academia, cannot return.

Theme 2.3: Promotes career exploration and planning (6 M). Faculty and administrative stakeholders noted the value of equipping pre- and post-doctoral researchers with a breadth of training beyond the academic career path, and that providing role models in different jobs allows current researchers to see themselves in those positions in the future. Fariba said PhD alumni, who are successful professionals, have mastered skill sets in their jobs, and hence hearing from them can add value to training and career exploration. These stakeholders also noted how mandatory Individual Development Plans (IDPs) set the framework for helping pre- and post-doctoral researchers organize their goals to obtain the skills necessary for those jobs.

Theme 2.4: Cycle of positive fulfilment for program (3 M). Individuals commented that future applications to the university will benefit from the existence of a good professional development

program, since many prospective students look for these opportunities. Fabio captured this sentiment, commenting that a professional development program is valuable for faculty to attract good people to the lab.

Opportunities to improve identified among faculty and administrators.

Theme 2.5: Tailored experience and exposure (17 M). Multiple comments were made about the need for students to be exposed to options early in graduate school. Fariba believes that graduate programs should encourage researchers to think about career planning before they start their graduate careers. It was also suggested that time commitment and type of engagement should align with educational stage. Others thought that for professional development activities to be efficient, it should be modular so the training experience can be tailored to each pre- and post-doctoral researcher's interests and can be prioritized individually.

Theme 2.6: Concerns and perceptions (13 M). Some faculty saw professional development as a waste of time that could lengthen time to degree completion. Finley's comment indicates the belief that pre- and post-doctoral researchers need to do one thing really well, even if only to focus on their dissertation research. Finley also believes that professional development, although important, should be done outside of research training, as researchers do need to do a lot of soul searching. Fariba and other faculty showed concern that smaller universities would not be able to launch their own professional development programs.

Theme 2.7: Narrow definition of professional development (6 M). Several of the more cautious and non-supportive participants interviewed thought that professional development only revolved around academic skills. Finley, for example, believes that research development is

career development. Furthermore, Finley believes that as educators, faculty and administrators should teach students to recognize their limits, and that their training is a privilege. These individuals did not indicate awareness that the phrase ‘professional development’ includes more than learning to publish, teach, and write grants.

Stakeholder 3 –External-facing Staff

Reasons to engage with industry identified by external-facing offices

Theme 3.1: Innovation and entrepreneurship (16 M). The interaction between industry and academia toward innovation and entrepreneurship is captured by Simha’s comment that she needs to bring the voice of the market to the university, helping to inform the direction of academic research to provide added value. The growth of biomedical technologies has spurred the enthusiasm to work with scientists to translate research and technologies. These offices work closely with researchers at the institutions, including pre- and post-doctoral researchers, as well as with industry representatives to evaluate the viability of projects.

Theme 3.2: Building partnerships (11 M). Examples of partnership benefits include fundraising, increasing awareness of internship programs, and involving industry in courses or events developed for pre- and post-doctoral researchers. Interactions with these groups leads to a better understanding of how small and large companies can better support initiatives such as providing hand-on experiences to these researchers. Shandra explained that these partnerships matter, as large companies can guide content creation of curriculum as well as provide longer-term help to universities through the development of ideas and resources. Soren encouraged the more holistic

approach, and coordinating with state-wide and economic development offices to create partnerships between academia and industry.

Theme 3.3: Fundraising or financial support (10 M). Shyla shared that another primary purpose for external-facing offices to interact with campuses is to create sponsored research. Interestingly, Shanice emphasized mutually beneficial goals leading to fundraising, where their external-facing office's motto is "time, talent, treasure".

Theme 3.4: Benefit to pre- and post-doctoral researchers (4 M). In these external-facing offices' views, the primary reason to engage with external stakeholders is to provide exposure. As Simha shared, she wants to expose researchers to perspectives other than her own, and the diversity of perspectives and options available on campus are enhanced by industry interactions.

Reasons external stakeholders engage with academia identified by external-facing staff

Theme 3.5: Early access to emerging technologies and innovations (18 M). A recurring theme centered on external stakeholders' keen interest in early access to emerging technologies or innovation. Simha commented on the interest, describing external stakeholders' intellectual curiosity, love of science, and being part of new discovery, while Saachi's opinion shared external stakeholders' interest in getting first access to emerging technologies developed in academia. Sree advocated for using industry partners' academic interests to incorporate experiential learning experiences within curricula.

Theme 3.6: Developing an entrepreneurial mindset (11 M). Another recurring theme was the goal of assisting in developing an entrepreneurial mindset, with Shandra's response capturing

why industry partners are interested in PhD level researchers – because people who are adaptable, flexible, and can work in teams are needed across the workforce. They recommended instilling an entrepreneurial mindset to make pre- and post-doctoral researchers more competitive. They also advocated that these skills can and should be taught to all students to better serve both themselves and the workforce more broadly, referring to a joint research brief on entrepreneurial mindset (Ernst & Young & Network for Teaching Entrepreneurship [4], and to reports published by the EU Commission [5] and World Economic Forum [6])

Theme 3.7: Faculty expertise or connections (8 M). External offices perceive faculty expertise or connectors as a strong motivator for external partners to interact with universities. Shandra indicated that companies can get advice on how to improve their businesses and smaller companies can connect with support services.

Theme 3.8: Talent identification (6 M). Unsurprisingly, external facing offices believe some external stakeholders view campuses as a talent identification hot-spot to build a workforce pipeline. Sahana points out that employers are seeking talent but are not interested in career fairs, implying that they would rather vet talent by getting to know the pre- and post-doctoral researchers in more organic ways than career fairs allow.

Theme 3.9: Provide scholarships/grants, or fund programs (5 M). External-facing staff believe that external partners are interested in funding research at a variety of levels, including generating revenue from patents or licensed products. Sven emphasized sponsored research programs as a way for companies to provide funding for universities through support of faculty projects.

Theme 3.10: Pay it forward (3 M). Some external-facing staff find that external partners are interested in helping in any way they can, in an effort to give back to institutions, while others find industry professionals are interested in helping, in order to be part of an exciting new professional development activity. Shanice mentioned that since external stakeholders understand what students need to be successful, they believe can help the next generation, and in fact wish that they had these types of programs when they were pre- and post-doctoral researchers.

Although not mentioned frequently enough to constitute a theme, a few internal-external partners mentioned that *prestige (2 M)* played a role in their engagement with academia. Some external partners are interested in developing relationships to create legacies in their names at the institutions. Saachi explained that external stakeholders feel they gain credibility by interacting with the university and being considered an asset.

External stakeholders' interest in STEM pre- and post-doctoral researchers identified by external-facing staff

Theme 3.11: Business experience and STEM knowledge (10 M). Among external-facing staff, there was a strong sentiment that business experience was more valuable than technical background. Shandra noted entrepreneurial mindset skills are important preparation for any career. Although STEM knowledge is important, the distinguishing feature for some companies is business acumen.

Theme 3.12: Expertise/talent pipeline (10 M). According to the external facing staff, one major benefit for external companies to interact with their office is access to new talent. Saachi

indicated that a side effect of technology application review is a connection with predoctoral researchers who are potential talent. The pre- and post-doctoral researchers who provide the expertise on collaborations and consultation are also possible future employees at these companies.

Theme 3.13: Mutual Benefit (10 M). The idea that pre- and post-doctoral researchers' relationship with other internal and external stakeholders is mutually beneficial was reflected by multiple stakeholders. Sahana noted that their institution's technology transfer internship program helps students get jobs, publicizes the university, and helps fulfil external companies' employment goals.

Theme 3.14: Support student needs (4 M). The general opinion is that external stakeholders have an interest in STEM pre- and post-doctoral researchers because they understand and support faculty and pre- and post-doctoral researcher needs. Shanice commented that they want to contribute to the pre- and post-doctoral researcher professional development and academic experience. They recall their own educational experience and reflect upon what is needed.

External-facing staff's additional thoughts

Theme 3.15: Specific advice to pre- and post-doctoral researchers (11 M). Many had suggestions for researchers to better prepare for their careers, but important advice was summed up best by Scott, who said that everyone should be prepared to learn on the job because the value of a PhD is that one can learn something new independently. Taking risks, trusting your critical thinking, and relying upon other transferrable skills developed in training, were strong messages shared by the external facing staff.

Theme 3.16: New perspectives on training needed (6 M). These internal stakeholders believe that to best prepare for any future career, it is helpful for pre- and post-doctoral researchers to expose themselves to many different experiences. Scott thinks it is important to gain skill sets to prepare for future interactions, no matter what career plan one has. Thus, embedding industry perspectives on workforce development into the curriculum can facilitate job seeking as well as establish long-term interactions between industry and academia.

Theme 3.17: Communication coordinator (4 M). Several external-facing staff thought that there should be a coordinated effort to engage with external partners. In particular, Shanice thought that their office of alumni engagement should be used to connect with alumni, and coordinate communication.

Theme 3.18: Alumni engagement role (3 M). Many external-facing office initiatives engage alumni, with a fundraising end-goal for institutions. On the other hand, alumni are interested in the prestige or ability to create a legacy, as well as to pay it forward to the next generation of pre- and post-doctoral researchers. Shanice shared that what appears important to each individual alumna/alumnus for engagement is being informed of the expected time commitment and financial request, and expect in return to have networking opportunities and to be connected to the right pre- and post-doctoral researcher for mentoring and/or hiring.

Stakeholder 4 – External Partners –Non-Profits/ Societies

External partner organizations' engagement with academia

Theme 4.1: Building relationships (16 M). Building relationships was a large motivator for societies, foundations and non-profits to interact with academia. Ellen sees the role of the non-

profits as the access point bringing academia and the private sector together. Forming bridges and connections is viewed as essential for the non-profit's missions.

Theme 4.2: Prestige, recognition, public visibility (8 M) While these external partners want to visit and interact with academia, they see it as a status-raising opportunity. Ellen says they are interested in the impact it can have on their organization, such as receiving public credit or being included as a full partner in endeavours. Non-profits are interested in benefits of prestige and visibility their organization could receive for such engagement.

Theme 4.3: Catalyst for connections, knowledge (5 M) Foundations and societies see themselves as a space for understanding commonalities and allowing for synergistic relationships to form. Eric captured this sentiment, commenting that their goal is to create a flow of knowledge between universities. This theme indicates that societies, non-profits, and funders may have more resources and opportunities available that are underutilized by universities.

Societies, foundations, and non-profits resources to offer

Theme 4.4: Expertise and advice (6 M) Societies and foundations have several scientists on staff possessing a wide range of expertise and perspectives. Ellen expressed that scientific staff should be tapped for their expertise, while Evan indicated they can provide the context for scientists to be understood by governmental agencies, as well as provide scientists context on how policies are made.

Theme 4.5: Online resources, guides (4 M). Societies, foundations and non-profit external stakeholders compile several online resources for wider use. A goal of these stakeholders is to

create and share among the community, and as Ellis stated, they hope more universities will engage with the resources they have compiled.

Theme 4.6: Experiential learning (4 M). A resource many institutions are looking for is experiential learning opportunities, and some societies, foundations, and non-profits offer this resource. Models include short-term fellowships, or longer period training mechanisms. Eric described a model within their organization as a mechanism to train leaders. Non-profits advise that pre- and post-doctoral researchers have a "growth mindset" and should use time in graduate school to test the waters to become a leader.

A topic that arose only infrequently was *self-exploration (2 M)*. An important aspect of career planning and exploration is the notion of understanding one's strengths and skills. Eric recommended that pre- and post-doctoral researchers make use of personality assessments, to build awareness of the skills they have that industry seeks.

Challenges faced by external partner organizations

Theme 4.7: Funding (4 M). Societies, foundations, and non-profits face funding limitations. Though they are keen to encourage careers at their organization, they may have limited resources for fellowships or research. Ellis described how the rate of funding they receive annually has been stagnant, and hence, unfortunately, fewer awards are given out each year. Importantly, these partners encourage that the affiliation between non-profits and academia should focus on relationships and resources, other than financial reliance.

Theme 4.8: Connect with target audience (3 M). External entities struggle with knowing whom to contact at institutions to ensure they are reaching the appropriate audience. Ebony recommended that universities have a one-stop shop, so external entities know whom to contact.

Theme 4.9: Flexible, creative models (3 M). Another challenge faced by these stakeholders is interacting within the strict design of educational programs at universities. They recommend flexibility to allow more exploration time for students; Ellis highlighted the need to propose different ways of training to prepare students for their futures, and to create and track these models with evidence-based outcomes and effectiveness research. Non-prescriptive models for graduate learning could open doors for more engagement with non-profits and foundations. Relatedly, Evan communicated the desire to talk to faculty about the importance of career exploration.

External partner organizations' view of career preparedness improvements

Theme 4.10: Develop and broaden skills, learning approach (11 M). The need for pre- and post-doctoral researchers to expand their skills was discussed frequently by external partners. Evan specified the importance of diversifying training to include non-science courses and to expand researchers' skills to include science communication and persuasive writing.

Theme 4.11: Take early initiative (8 M). Many external partners stressed the need for pre- and post-doctoral researchers to take initiative early to create opportunities and explore their options. Ellen voiced the advice succinctly when she advocated for researchers to get involved in an organization and develop relationships well in advance of their job search.

Theme 4.12: Improve self-efficacy and growth mindset (6 M). Societies, foundations and non-profits encourage pre- and post-doctoral researchers to adopt a growth mindset, and to be aware of demand for their skills. Ebony impressed upon not constraining oneself, and to explore and grow with one's science and research. Others touched upon the necessity to acknowledge how opportunities offered by societies are equally important and valuable to research internships.

Theme 4.13: Listen with humanity, broaden diversity (4 M). A theme emerged from multiple conversations that being an inclusive leader is crucial, and having that outlook is important for pre- and post-doctoral researchers. Eric mentioned the need to listen to the majority and also historically under-represented minority groups, while Emily discussed the need to learn how to support underrepresented groups to help the next generation of scientists. Ellen highlighted the need to pause and learn about others, and to not always center one's conversation around an individual's point of view.

Although mentioned infrequently among these participants, and hence not classified as a theme of its own, a key concept to finding the right career path is *networking and engagement opportunities (2 M)*; Ebony advised pre- and post-doctoral researchers find ways to connect beyond conferences such as SACNAS, and suggested the need for regional conferences centered around graduate careers to help expose pre- and post-doctoral researchers to opportunities.

Some pieces of advice were provided to researchers, such as *get involved in professional societies (1 M)* to expand their spheres and take on leadership roles. Moreover, *defining success (1 M)* of the relationship should be left up to the fellows or pre- and post-doctoral researchers themselves since they know what metrics are important to them. As Ebony advised, fellows should learn to identify their own success measures.

"Definition of success is defined by the individual; the PhD is a key to the door but what door do you want to open?"

A few individuals mentioned that they did not want to come to campus but rather wanted to *take the pre- and post-doctoral researchers off campus (2 M)* to experience advocacy in the government or how their non-profit operates in the real world.

External partners' view of engaging with academic institutions

Theme 4.14: Invitations (5 M). These non-profit and society stakeholders are keen to engage and to disseminate their resources. As Ellis highlighted, these organizations are accessible, and they encourage universities to invite them to visit campus and coordinate their visits. Their view is that universities should reach out and contact the non-profits rather than solely relying on the reverse.

Another theme included inviting researchers off campus (2 M).

A few varied challenges were identified infrequently by external partner organization stakeholders but should be taken into consideration. One challenge identified was that an organization's (e.g. non-profit) *offerings should be integrated* into training *(2 M).* Although flexibility is necessary as mentioned above, some participants were keen, and even preferred to embed their resources in the graduate curriculum, to ensure all graduate student pre- and post-doctoral researchers have access. External partners wish to *engage at all levels with academic institutions (2 M).* Strategic planning requires engagement by all members of an academic institution; hence societies, foundations, and non-profits must prioritize the amount of engagement necessary at each level, to achieve their goals, and institutions must be willing to

engage. An external partner mentioned the challenge that the *wrong people are making decisions (1 M)* at some of these external organizations, and it appeared that once PhD-level employees were involved in the decision-making process, the offerings they provided to academia were much more relevant. Finally, another challenge faced by external partners is the need for more industry mentors. Ebony describes the difficulty in getting enough mentors for the fellows program at their organization.

Stakeholder 5 – External Employers -Industry

Reasons why industry external stakeholders engage with academia

Theme 5.1: Recruiting and broadening their reach (8 M). Companies are interested in being involved in professional development programs. As Iris explained, these programs are a pipeline into the company, even if the company is small and only a few PhDs are hired. Some companies see a relationship with a university as a geographically local recruitment tool.

Theme 5.2: Building long-term relationships (5 M). Shandra shared that the same person from the company went to an institution every year, thereby creating a strong connection. She believes that this helps develop a sense of trust and community, allowing these representatives to then serve as technical recruiters to help pre- and post-doctoral researchers prepare for interviews. When an alumna/us acts as the liaison, there is an added benefit of helping maintain connections and creating a sense of community.

Theme 5.3: Bidirectional partnerships (4 M). Some employers even rely on universities to facilitate their research and development. As Ira pointed out, pre- and post-doctoral researchers

generate early discovery and pre-clinical data. Companies see this as a two-way relationship that benefits both parties.

Theme 5.4: Advisory and feedback roles (3 M). Relationships between academic institutions and companies create and strengthen connections between the two. For example, Ira suggested industry can conduct focus groups to learn how to better tailor offerings such as internships to students, and while Ivy commented that industry can, and sometimes does, share with faculty how skills transfer to industry.

Training and collaboration industry-academia partnership benefits

Theme 5.5: Alumni and mentoring (8 M). The modes of interaction between industry and academia include alumni working with their *alma mater*. Of note, Ivan indicated that the personal recruiting via doctoral alumni is not used enough. Other interactions, such as pre- and post-doctoral researcher outreach, were touched upon. For example, Ivan provided feedback that their company appreciates it when these researchers initiated relationships.

Importantly, if a company's needs are already met, they admitted to having no motivation to interact with academia, as they believe that their reputation alone was sufficient to draw job applicants.

Theme 5.6: Programs and industry expertise (6 M). Irina pointed out that industry has efficient resources to attack bigger problems, where academia has complementary skills for broad questions. Ian reiterated the need for increased awareness of industry operations, such as drug development and marketing.

Theme 5.7: Learn industry-relevant skills (4 M). External stakeholders shared an important opinion: they do not think that the graduate school curriculum prepares pre- and post-doctoral researchers sufficiently for industry since the goals of research are different in the two sectors. Ira emphasized this idea, saying academics must learn how to better design experiments of relevance to industry. Since the goal of biotech is to commercialize rather than publish, experimental criteria are very different.

Theme 5.8: Recalibrate importance (3 M). Industry and academia place weight on different metrics and therefore pre- and post-doctoral researchers need to shift their focus when applying for jobs. Iris bluntly stated that not all individuals in industry value publications when vetting new hires. Instead pre- and post-doctoral researchers should focus on how the transferrable skills they have can help the company move a product through the pipeline which is what is important to industry.

Theme 5.9: Grants and academic collaborations (3 M). Irina keenly noted that industry is to be viewed as a collaborator, and not a piggy bank. Industry employs top scientific minds, who are eager to collaborate with academia. Their goal, however, is not to help solve problems in research, but rather to advance their product to commercialization.

Differing priorities and organizational complexities – challenges with industry

Theme 5.10: Differing values (5 M). Ira believes that academia should welcome collaborations and not distrust industry standards as industry is highly regulated. Furthermore, Ira advised that academics should lose their negative attitude toward non-technical roles. Scientists who do go

down this path should be equally celebrated as successful. The different value systems between industry and academia should not create a level of disdain between the two.

Theme 5.11: Point of contact (3 M). Ian pointed out that the relationship between industry and academia needs longevity and suggested that universities should identify an individual program director who assigns or recruits students. The complexity of the organizations often creates difficulties for either party (companies and universities) to know who the appropriate point person is in the other party. Relatedly, to maintain longevity, Ian also impressed upon the importance of tracking the impact of an interaction or event.

External industry employers' views on challenges with pre- and post-doctoral researcher preparedness

Theme 5.12: Understanding options, industry culture and priorities (8 M). Industry professionals' advice to pre- and post-doctoral researchers is, as Ivan shared, the importance of understanding what an industry job entails and what the job title means. Imani stated that observation or hands-on experience is critical before applying.

Theme 5.13: Develop communication skills (8 M). A frequently mentioned theme highlighted the need for pre- and post-doctoral researchers to develop good communication skills before embarking on a career in industry. Imani stressed upon the need to better synthesize information and communicate complicated topics in an exciting and concise way.

Theme 5.14: Present experience and motivation (7 M). While it is well-acknowledged that PhD-trained individuals have good training and experience, it is important to be able to translate this appropriately. Irina advised that these researchers need to understand the problem-solving

process is valuable, and that they should not panic about what they don't know in the industry setting.

Theme 5.15: Relationship building and collaborations (5 M). Collaborations are seen as essential, and hence the ability to build and sustain relationships is critical to succeeding in industry. Ivy emphasized the need to help PhDs understand the collaborative nature of industry research. Additionally, Ira was quick to point out that each industry employee is of equal value: marketing managers and scientists are equally important team members.

Theme 5.16: Faculty culture change (4 M). An oft-thought opinion was voiced by Ivan when he commented that students shouldn't feel like they are operating behind their advisor's back. To aid in faculty culture change, Ivy advised that CPD practitioners share with faculty how skills transfer to industry and offer perspectives. Irina, aware of management hierarchies and dynamics, advised making changes from the top, by approaching department chairs or deans to be open-minded to industry career outcomes for their graduates. Faculty culture needs to change to accept different career paths.

How to encourage industry professionals to interact and visit campus

Theme 5.17: Invitations (6 M). Several companies are glad to visit or interact with academic institutions. Ivy highlighted that they do this when students invite them for career panels, informational interviews, campus visits, or to set up site visits. Simply being asked to be on a career panel or other programs is sufficient.

Theme 5.18: High-impact events (3 M). Iris described how more comprehensive engagement with faculty during their visits (e.g., larger events can involve faculty beyond the event, networking opportunities, informal interactions, one-on-one meetings) helps make industry partners feel welcome. Industry has interest if a university hosts events that guarantee large pre- and post-doctoral researcher attendance, so they have maximal impact for their visit.

Theme 5.19: Match-ups (3 M). Ian mentioned the value of listservs or student groups who are interested in various companies' work, as these lists can be shared with appropriate companies as a recruiting tool. Companies would be interested in interacting if they are assured to find recruits who match up as the appropriate kind of candidate.

Theme 5.20: Open-minded to industry (3 M). While culture change is important, issues pertaining to intellectual property constraints, or access to departments involved in translational research were described as a challenge.

A few other comments represented important opinions, but were not repeated across interviews, and are hence not classified as themes. A participant described they had no desire to visit campus, but instead preferred to host site visits for pre- and post-doctoral researchers. Another described limited bandwidth at their organization to devote time and resources to university relations.

References:

1. Sherrer KJ, Prelip ML. A Multifaceted Approach to Public Health Career and Professional Development Training. Health Promot Pract. 2019;20(6):932–40.

2. Bixenmann R, Natalizio BJ, Hussain Y, Fuhrmann CN. Enhancing Dissemination of Evidence-Based Models for STEM PhD Career Development; a Stakeholder Workshop Report. Worcester, MA; 2020.

3. Van Doren D, Corrigan HB. Designing a Marketing Course With Field Site Visits. J Mark Educ [Internet]. 2008 May 19;30(3):189–206. Available from: https://doi.org/10.1177/0273475308318071

4. Ernst & Young, Network for Teaching Entrepreneurship (NFTE). Supporting the next generation: The entrepreneurial mindset and the future of work [Internet]. 2018. Available from: https://www.nfte.com/wp-content/uploads/2020/07/Joint-Research-Brief-The-Entrepreneruial-Mindset-and-the-Future-of-Work.pdf

5. Bacigalupo M, Kampylis P, Punie Y, Van Den Brande L. EntreComp: The Entrepreneurship Competence Framework. 2016.

6. Drexler M, Eltogby M, Foster G, Shimizu C, Ciesinski S, Davila A, et al. Entrepreneurial Ecosystems Growth Dynamics – the Early-Stage Company Around the Globe and Entrepreneur's Perspective [Internet]. World Economic Forum. 2014. Available from: www.weforum.org
