# Supplementary material for: Using stakeholder insights to enhance engagement in PhD professional development
Source: PLoS One. 2022 Jan 27;17(1):e0262191. doi: 10.1371/journal.pone.0262191 (PMC8794081; doi:10.1371/journal.pone.0262191)
Supplement: S4 File — (PDF) [file pone.0262191.s006.pdf]

S4 File: Examples for Diversifying Networks

Consider diversifying one's network by intentionally paying attention to:

1. Geographic location
2. Country of origin
3. Gender
4. Race/Ethnicity, especially BIPOC
5. LGBTQIA+
6. Training speciality (e.g. STEM, Humanities, Social Sciences)
7. Sector (Business, Government, Non-profit)
8. How you know them (work, societies/organizations, religious activities, sports teams, neighbourhood, etc.)
9. Level of support for your activities
