# Supplementary material for: Using stakeholder insights to enhance engagement in PhD professional development
Source: PLoS One. 2022 Jan 27;17(1):e0262191. doi: 10.1371/journal.pone.0262191 (PMC8794081; doi:10.1371/journal.pone.0262191)
Supplement: S6 File — (PDF) [file pone.0262191.s008.pdf]

S6 File: Common themes across and within stakeholder groups from underlying data

COMMON THEMES ACROSS STAKEHOLDERS

- Distinct need for Career & Professional Development
- Importance of networks, partners, and collaborations
- Timing and content of programming
- Expanding purview of scientific/academic training
- Identifying and creating streamlined access
- Focus on aligning CPD programming with needs
- Tailored experiences for populations
- Normalizing and expanding definitions of CPD activities

THEMES WITHIN STAKEHOLDER GROUPS

TRAINEES

Benefits

- Efficiency, productivity, and content
- Networking, community, role models
- Exposure and decision-making of career paths
- Embedded/required
- Prestige-recruitment

Challenges/Opportunities to improve

- Consistent exposure throughout training
- Centralized/access
- Growth/challenges
- Faculty permission

FACULTY/ADMINISTRATORS

Benefits

- Evolving training requirements and climate
- Awareness of workforce and outcomes
- Promotes career exploration and planning
- Cycle of positive fulfillment for program

Challenges/Opportunities to improve

- Tailored experience and exposure
- Concerns and perceptions
- Narrow definition of 'professional development'

INTERNAL-EXTERNAL FACING

Purpose of engagement

- Innovation and entrepreneurship
- Building partnerships
- Fundraising or financial support
- Benefit to trainees

External stakeholder interests

- Early access to emerging technologies
- Developing an entrepreneurial mindset
- Faculty expertise/connections
- Talent identification
- Provide scholarship/grants/fund programs
- Pay it forward

Interest in STEM trainees

- Business experience and STEM knowledge
- Expertise/talent pipeline
- Mutual benefit
- Support student needs

Other: thoughts/advice

- Specific advice to trainees
- New perspectives in training needed
- Communication coordinator
- Alumni engagement role

EXTERNAL: FOR PROFIT

Purpose of engagement

- Recruiting and broadening their reach
- Building long-term relationships
- Bidirectional partnerships
- Advisory and feedback roles

Models and Resources

- Alumni and mentoring
- Programs and industry experience
- Learn industry-relevant skills
- Recalibrate importance
- Grants and academic collaborations

Challenges

- Differing values
- Point of contact

Advice and challenges for trainees

- Understanding options, industry culture/priorities
- Develop communication skills
- Present experience and motivation
- Relationship-building and collaborations
- Faculty culture change

Reasons to visit/interact

- Invitations
- High-impact events
- Match-ups
- Open-minded to industry

EXTERNAL: NON-PROFIT

Purpose of engagement

- Building relationships
- Prestige, recognition, public visibility
- Catalyst for connections, knowledge

Resources to offer

- Expertise and advice
- Invite and coordinate visits
- Online resources/guides
- Experiential learning

Challenges for the stakeholder

- Funding
- Connect with target audience
- Flexible, creative models

Opportunities for trainees

- Develop and broaden skills
- Take early initiative
- Improve self-efficacy, growth mindset
- Listen with humanity, broaden diversity

Reasons to visit/interact

- Invitations
